# Development of a nomogram for predicting clinical outcome in patients with angiogram‐negative subarachnoid hemorrhage

**DOI:** 10.1111/cns.13712

**Published:** 2021-07-28

**Authors:** Anke Zhang, Zeyu Zhang, Wen‐Bo Zhang, Xiaoyu Wang, Cameron Lenahan, Yuanjian Fang, Yujie Luo, Yibo Liu, Shuhao Mei, Sheng Chen, Jianmin Zhang

**Affiliations:** ^1^ Department of Neurosurgery The Second Affiliated Hospital School of Medicine Zhejiang University Zhejiang China; ^2^ Department of Neurosurgery National Clinical Research Center for Child Health The Children's Hospital of Zhejiang University School of Medicine Zhejiang China; ^3^ Center for Neuroscience Research Loma Linda University School of Medicine Loma Linda CA USA; ^4^ Burrell College of Osteopathic Medicine Las Cruces NM USA

**Keywords:** angiogram‐negative subarachnoid hemorrhage, nomograms, outcome research, prognosis

## Abstract

To the best of our knowledge, this is the largest clinical retrospective study in AN‐SAH patients, and is the first time to establish accurate predictive models paired with bleeding pattern.

**Background:**

Angiogram‐negative subarachnoid hemorrhage (AN‐SAH) has a definite incidence of delayed cerebral ischemia (DCI) and poor clinical outcomes. The purpose is to screen independent factors and establish a nomogram to guide the clinical therapy and assess post‐discharge prognosis.

**Methods:**

We identified 273 consecutive patients referred to our institute from 2013 to 2018 for AN‐SAH. A nomogram to predict poor outcomes was formulated based on the multivariable models of independent risk factors. The accuracy and discrimination of nomograms were determined in training and internal validation cohorts.

**Results:**

The overall poor outcome rates of AN‐SAH were 14.3% and 8.7% at 3 months and 12 months, respectively. In addition, perimesencephalic AN‐SAH (PAN‐SAH) presented with a more unfavorable prognosis compared with non‐perimesencephalic AN‐SAH (NPAN‐SAH). The clinical prognosis was associated with the World Federation of Neurosurgical Societies scale (WFNS) (odds ratio, 3.82 [95% CI, 1.15‐12.67] for 3‐month outcome; and odds ratio, 31.69 [95% CI, 3.65‐275.43] for 12‐month outcome), Subarachnoid hemorrhage Early Brain Edema Score (SEBES) (odds ratio, 10.39 [95% CI, 1.98‐54.64] for 3‐month outcome; odds ratio, 10.01 [95% CI, 1.87‐53.73] for 12‐month outcome), and symptomatic vasospasm (odds ratio, 3.16 [95% CI, 1.03‐9.70] for 3‐month outcome; odds ratio, 5.15 [95% CI, 1.34‐19.85] for 12‐month outcome). The nomogram was constructed based on the above features, which represented great predictive value in clinical outcomes.

**Conclusions:**

Symptomatic vasospasm, high WFNS, cerebral edema, and NPAN‐SAH after hemorrhage were associated with poor outcome of AN‐SAH. The nomogram with WFNS (3‐5), SEBES (3‐4), vasospasm, and NPAN‐SAH represented a practical approach to provide individualized risk assessment for AN‐SAH patients.

## Graphical abstract

To the best of our knowledge, this is the largest clinical retrospective study in AN‐SAH, and is the first time to establish accurate predictive models paired with bleeding pattern.

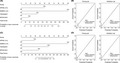

## 1 INTRODUCTION

Subarachnoid hemorrhage (SAH) is an uncommon and severe subtype of stroke, accounting for 5% of the patients with stroke. The rupture of an intracranial aneurysm is the underlying cause in the majority of cases.^1^ However, in approximately 15% of SAH cases, vascular abnormalities are not demonstrated through vascular imaging or digital subtraction angiogram (DSA), a condition referred to as AN‐SAH.^2^ AN‐SAH is a unique variant of SAH, for which no structural aberration can be found as a primary cause for spontaneous hemorrhage.^2^, ^3^ AN‐SAH can be categorized as PAN‐SAH or NPAN‐SAH according to the bleeding patterns.^4^ Half of the patients with AN‐SAH were presented with the perimesencephalic type, which is known to present better clinical outcomes and lower rates of vasospasm than those observed with aneurysmal SAH.^5^ However, AN‐SAH still accompanied definite incidence of DCI and poor clinical outcomes, especially in patients with the non‐perimesencephalic bleeding pattern.^6^

So far, more than 40 clinical grading scores have been proposed to assess the clinical severity after SAH, including the Hunt‐Hess (HH), Subarachnoid hemorrhage Early Brain Edema Score (SEBES), Glasgow Coma Score (GCS), the World Federation of Neurosurgical Societies scale (WFNS), and the modified Fisher Scale (mFS).^7^, ^8^, ^9^, ^10^, ^11^, ^12^, ^13^ These prognostic grading scores were not initially designed for AN‐SAH patients, but were also commonly used in the clinical assessment and prognostic prediction of AN‐SAH patients due to the lack of standardized evaluation indicators. In our previous study, we compared the prognostic value of several clinical scores in AN‐SAH patients, and demonstrated their favorable adaptation to this type.^5^ However, we did not distinguish the difference between PAN‐SAH and NPAN‐SAH, or did we evaluate other significant factors associated with outcomes. Until now, the effectiveness of clinical, radiological, or combined scores had rarely been analyzed and validated in AN‐SAH patients. Furthermore, the small sample size of included patients and the lack of further assessment of bias due to individual interpretation of AN‐SAH may skew the results.^6^

An optimal selection of grading scales may assist clinicians in making informed decisions, and allow for improved care and effective treatment in AN‐SAH patients. Herein, the aim of this study is to screen out independent associated factors, emphasize optimal grading scores, and establish a nomogram to guide the clinical therapy and assess post‐discharge prognosis.

## 2 METHODS

### 2.1 Study population and follow‐up

A retrospective study was conducted to identify all consecutive patients who were referred to our institute and diagnosed with AN‐SAH between January 2013 and December 2018. This study included past clinical records and imaging data of 1,219 consecutive patients with spontaneous SAH. The demographic, clinical, and imaging data were reviewed. The inclusion criteria utilized are listed as follows: (1) patients were screened by computed tomography angiography (CTA) at admission, followed by emergent DSA within 72 hours, and diagnosed with AN‐SAH; and (2) patients with a follow‐up period > 12 months after discharge. Exclusion criteria encompassed: (1) patients with a history of trauma or previous brain injury; (2) patients who had serious comorbidities prior to SAH onset; and (3) patients with unavailable radiological images or missing follow‐up data. A total of 35 patients were excluded, leaving 273 patients eligible for final analysis (Figure S1). The Institutional Review Board approved all aspects of this study and waived informed consent.

### 2.2 Patient management and grading

All patients were managed according to the Neurocritical Care Society and American Heart Association SAH guidelines.^14^, ^15^ Patients were administered nimodipine to prevent vasospasm, and euvolemia was maintained via intravenous hydration. Baseline features, such as age, gender, social history, and medical history, were all collected. The SAH severity was assessed through clinical grading and radiological images during admission. The clinical assessment included the GCS, HH grade, WFNS, mFS, SEBES, and intraventricular hemorrhage (IVH) through image valuation. Scores ranging from 3 to 5 for WFNS and HH, and 3 to 4 for mFS and SEBES, were considered high grade. The bleeding pattern criteria were defined according to a previous study as PAN‐SAH and NPAN‐SAH.^16^

SAH‐related complications, including symptomatic vasospasm (new focal neurological signs or deterioration of consciousness, excluding other definitive causes),^17^ delayed cerebral infarction (new infarction on radiological imaging), rebleeding (new or expanded hemorrhage on CT), encephaledema, and seizure. Symptomatic vasospasm and delayed cerebral infarction were defined as delayed cerebral ischemia (DCI).^18^, ^19^, ^20^ Clinical prognosis was assessed using the modified Rankin Scale (mRS) at 3 months and 12 months following discharge, which was recorded by outpatient records and telephone interview. According to the mRS, the prognosis was categorized as either poor outcome (mRS 2‐6) or favorable outcome (mRS 0‐1).

### 2.3 Nomogram construction and validation

The patients were randomly divided into a training cohort and a validation cohort using a random number list generated by SPSS. In our study, we selected one half of the patients as the training group, and the remaining patients as the validation group, so that the clinical characteristics except the four independent factors showed no significance between these two sets, and aimed to emphasize predictive value of nomogram model with more accuracy. We analyzed and compared the general clinicopathological data of the two cohorts. The factors with *P* value less than 0.05 in multivariated analysis were selected as predictors in nomogram. In addition, due to the significant difference of clinical progression and prognosis between PAN‐SAH and NPAN‐SAH, the factor of NPAN‐SAH was also considered as a label in the nomogram. Based on these factors, the R software version 2.13.2 (https://www.r‐project.org) generated nomogram models to predict post‐discharge outcomes for patients at 3 and 12 months. The performance evaluation of the nomogram includes discrimination, calibration curves, and ROC curve, which were verified by the calibration curve generated by the validation cohort.

### 2.4 Statistical methods

The continuous variables were reported as Mean±SD. For the clinical and neuroradiological data, patients with favorable or unfavorable outcomes were compared using descriptive statistics, Student’s *t‐*test for continuous variables, and χ^2^ or fisher exact test for categorical variables.

Both univariate and multivariable logistic regression were performed to estimate the risk of disability (mRS score ≥2 at treatment‐free follow‐up). We used a multivariable model with a forward stepwise regression procedure to screen out the potential predictors for poor outcome. The performance and accuracy of the nomogram was evaluated with the calibration and ROC curves to measure internal calibration and discriminative ability.

All analyses and graphical representations were performed with IBM‐SPSS V24.0 (SPSS Inc., Armonk, NY), Prism 8 (GraphPad Software, Inc.), and R Version 3.6.3 GUI 1.70 (The R Foundation for Statistical Computing). Statistical significance was set at *P* < 0.05.

## 3 Results

### 3.1 Demographics and clinical characteristics of the study population

The demographic features and clinicopathological characteristics of patients with PAN‐SAH and NPAN‐SAH are presented in Table S1. A total of 273 patients confirmed with AN‐SAH at admission were collected and analyzed. Detailed information on baseline characteristics, hospital complications, and clinical outcomes are summarized. There were 184 (67.4%) patients with PAN‐SAH and 89 (32.6%) patients with NPAN‐SAH identified. In the PAN‐SAH group, there were 90 women and 94 men, with a mean age of 55.7 ± 10.7 years. Among them, 37.5% and 34.2% have a history of drinking and smoking, respectively. Moreover, 34.2% and 7.1% of the PAN‐SAH patients have comorbidities of hypertension and diabetes, respectively. In the NPAN‐SAH group, there were 35 women and 54 men, with a mean age of 57.4 ± 11.8 years. As depicted in Table 1, the demographic data showed no statistical significance between the PAN‐SAH and NPAN‐SAH groups, including BMI. However, hospital complications, including symptomatic vasospasm, delayed cerebral infarction, rebleeding, and encephaledema, occurred more frequently in the NPAN‐SAH type (all *P* < 0.05), with the exception of seizure (*P* = 0.249). In addition, greater SAH severity (characterized by clinical data and radiological images) and poorer outcomes (assessed due to mRS) were observed in patients with NPAN‐SAH (*P* < 0.05).

**TABLE 1:** Comparison of Clinical characteristics, assessment, and complications between favorable and unfavorable outcomes in PAN‐SAH and NPAN‐SAH

	3‐month outcome						12‐month outcome					
	PAN‐SAH (n = 184)			NPAN‐SAH (n = 89)			PAN‐SAH (n = 184)			NPAN‐SAH (n = 89)		
	Favorable (n = 171)	Unfavorable (n = 13)	*P* value	Favorable (n = 63)	Unfavorable (n = 26)	*P* value	Favorable (n = 180)	Unfavorable (n = 4)	*P* value	Favorable (n = 70)	Unfavorable (n = 19)	*P* value
Gender (female)	83 (48.5%)	7 (53.8%)	0.712	26 (41.3%)	9 (34.6%)	0.559	89 (49.4%)	1 (25.0%)	0.621	31 (44.3%)	4 (21.1%)	0.066
Age	55.7 ± 10.6	55.4 ± 12.2	0.922	56.4 ± 12.0	59.7 ± 10.9	0.230	55.7 ± 10.8	56.6 ± 7.7	0.876	56.6 ± 11.8	60.4 ± 11.5	0.215
Drink	66 (38.6%)	3 (23.1%)	0.377	25 (39.7%)	9 (34.6%)	0.655	68 (37.8%)	1 (25.0%)	0.999	26 (37.1%)	8 (42.1%)	0.693
Smoke	59 (34.5%)	4 (30.8%)	0.999	21 (33.3%)	11 (42.3%)	0.422	61 (33.9%)	2 (50.0%)	0.607	23 (32.9%)	9 (47.4%)	0.242
Hypertension	58 (33.9%)	4 (30.8%)	0.934	20 (31.7%)	14 (53.8%)	0.051				24 (34.3%)	10 (52.6%)	0.144
diabetes	12 (7.0%)	1 (7.7%)	0.999	5 (7.9%)	4 (15.4%)	0.289	13 (7.2%)	0 (0%)	0.999	5 (7.1%)	4 (21.1%)	0.093
BMI			0.461			0.184			0.751			0.158
I < 18.5	8 (4.7%)	0 (0%)		1 (1.6%)	2 (7.7%)		8 (4.4%)	0 (0%)		1 (1.4%)	2 (10.5%)	
II 18.5‐23.9	85 (49.7%)	5 (38.5%)		31 (49.2%)	10 (38.5%)		87 (48.3%)	3 (75.0%)		34 (48.6%)	7 (36.8%)	
III 24‐27.9	71 (41.5%)	8 (61.5%)		29 (46.0%)	11 (42.3%)		78 (43.3%)	1 (25.0%)		32 (45.7%)	8 (42.1%)	
IV >28	7 (4.1%)	0 (0%)		2 (3.2%)	3 (11.5%)		7 (3.9%)	0 (0%)		3 (4.3%)	2 (10.5%)	
GCS <15	25 (14.6%)	4 (30.8%)	0.128	16 (15.4%)	20 (76.9%)	**<0.0001**	26 (14.4%)	3 (75.0%)	**0.013**	20 (18.6%)	16 (84.2%)	**<0.0001**
WFNS (3‐5)	2 (1.2%)	4 (30.8%)	**<0.0001**	8 (12.7%)	16 (61.5%)	**<0.0001**	3 (1.7%)	3 (75.0%)	**<0.0001**	10 (14.3%)	14 (73.7%)	**<0.0001**
HH (3‐5)	6 (3.5%)	2 (15.4%)	0.101	10 (15.9%)	16 (61.5%)	**<0.0001**	7 (3.9%)	1 (25.0%)	0.164	14 (20.0%)	12 (63.2%)	**<0.0001**
mFS (3‐4)	4 (2.3%)	1 (7.7%)	0.31	39 (61.9%)	19 (73.1%)	0.314	5 (2.8%)	0 (0%)	0.999	43 (61.4%)	15 (78.9%)	0.184
SEBES (3‐4)	1 (0.6%)	1 (7.7%)	0.137	2 (3.2%)	13 (50.0%)	**<0.0001**	1 (0.6%)	1 (25.0%)	**0.043**	4 (5.7%)	11 (57.9%)	**<0.0001**
IVH	24 (14.0%)	2 (15.4%)	0.999	21 (33.3%)	14 (53.8%)	0.072	25 (13.9%)	1 (25.0%)	0.458	24 (34.3%)	11 (57.9%)	0.062
Vasospasm	13 (7.6%)	5 (38.5%)	**0.004**	26 (41.3%)	23 (88.5%)	**<0.0001**	15 (8.3%)	3 (75.0%)	**0.003**	30 (42.9%)	19 (100.0%)	**<0.0001**
DCI	5 (2.9%)	4 (30.8%)	**0.002**	10 (15.9%)	17 (65.4%)	**<0.0001**	6 (3.3%)	3 (75.0%)	**<0.0001**	14 (20.0%)	13 (68.4%)	**<0.0001**
Rebleeding	0 (0%)	1 (7.7%)	0.071	0 (0%)	6 (23.1%)	**<0.0001**	0 (0%)	0 (0%)	0.999	0 (0%)	6 (31.6%)	**<0.0001**
Encephaledema	3 (1.8%)	0 (0%)	0.999	7 (11.1%)	11 (42.3%)	**<0.0001**	3 (1.7%)	0 (0%)	0.999	9 (12.9%)	9 (47.4%)	**0.001**
Seizure	1 (0.6%)	0 (0%)	0.926	0 (0%)	2 (7.7%)	0.083	1 (0.6%)	0 (0%)	0.999	0 (0%)	2 (10.5%)	**0.044**

Bold values are statistically significance.

**FIGURE 1:**
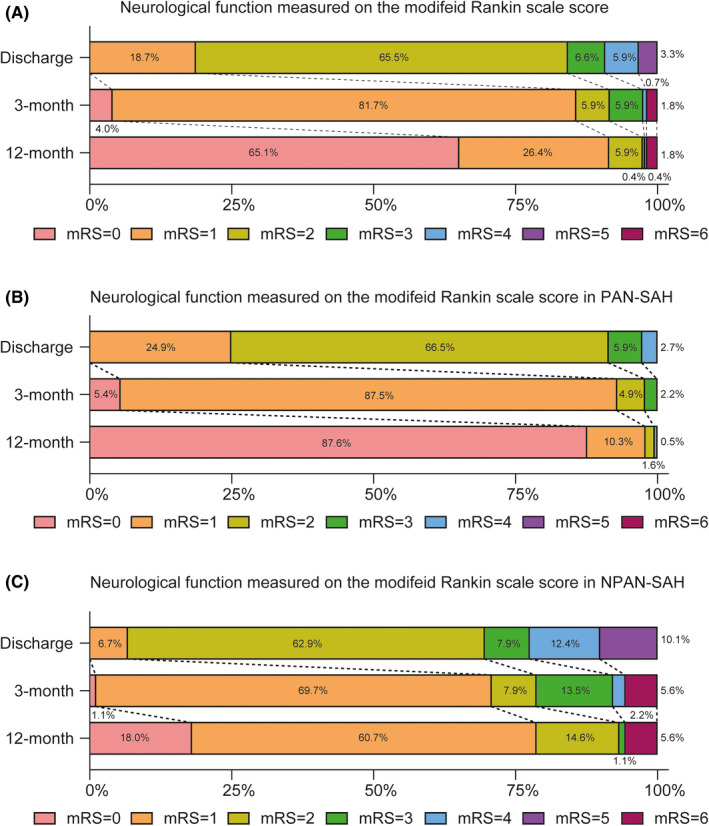
Neurological Function Measured on the Modified Rankin Scale Score for AN‐SAH Patients with Different Bleeding Patterns. The modified Rankin Scale Score distribution in overall patients (A), PAN‐SAH patients (B), and NPAN‐SAH patients (C). Data are provided from disposition, 3‐month, and 12‐month follow‐up

Of all the AN‐SAH patients, the majority of patients (81.3%) with mRS greater than 2 points were observed at discharge, of which poor outcomes were improved after 3 and 12 months with 14.3% and 8.5%, respectively (Figure 1A). In addition, there were 5 incidents of death, accounting for 1.8% of all cases. The distribution of patient outcome in PAN‐SAH demonstrated more favorable outcomes, with 7.1% and 2.1% at 3 months and 12 months, respectively. However, the proportion of poor outcome are 29.2% and 21.3% in the patients with NPAN‐SAH, respectively. It should be noted that these five cases of death were all identified as NPAN‐SAH, which accounted for 5.6% of the total NPAN‐SAH patients (Figure 1B and C).

### 3.2 Factors associated with poor outcomes in PAN‐SAH and NPAN‐SAH

According to the significant difference found in outcomes between the two bleeding patterns, we screened the factors associated with favorable outcomes in PAN‐SAH and NPAN‐SAH. Regarding SAH severity, disturbance of consciousness (GCS<15), high WFNS, high HH, and high SEBES all correlated with unfavorable outcomes at 3 months in the NPAN‐SAH type (all *P* < 0.0001), as well as the occurrence of complications, including symptomatic vasospasm, delayed cerebral infarction, rebleeding, and encephaledema (all *P* < 0.0001). However, only WFNS (*P* < 0.0001), symptomatic vasospasm (*P* = 0.004), and delayed cerebral infarction (*P* = 0.002) are related to the 3‐month outcome in PAN‐SAH patients. Besides, similar results were observed in 12‐month outcomes. As depicted in Table 1, except for the above factors, the occurrence of seizure (*P* = 0.044) is also related to 12‐month outcome of patients with NPAN‐SAH, while disturbance of consciousness (*P* = 0.013) is also related to 12‐month outcome in PAN‐SAH.

### 3.3 Univariate and multivariate analysis of risk factors associated with 3‐Month and 12‐month prognosis

To screen the common predictors, we selected the dominant factors, including disturbance of consciousness (GCS<15), WFNS (3‐5), HH (3‐5), SEBES (3‐4), symptomatic vasospasm, and delayed cerebral infarction, which can predict at least three situations for the following analysis. As shown in Table 2, combined with univariate analysis and multivariable logistic regression, the association between 3‐month disability and high WFNS (OR=3.82, 95% CI: 1.15‐12.67), high SEBES (OR=10.39, 95% CI: 1.98‐54.64), and symptomatic vasospasm (OR=3.16, 95% CI: 1.03‐9.70) remained significant in the forward stepwise multivariable model. Notably, high WFNS (OR=31.69, 95% CI: 3.65‐275.43), high SEBES (OR=10.01, 95% CI: 1.87‐53.73), and symptomatic vasospasm (OR=5.15, 95% CI: 1.34‐19.85) were the remaining risk factors for predicting 12‐month disability. Although the univariate analysis suggested that disturbance of consciousness, HH, and delayed cerebral infarction were predictors for disability, they were not evident in the last model. Interestingly, WFNS, SEBES, and symptomatic vasospasm are the common independent predictors for both the 3‐month and 12‐month prognoses.

**TABLE 2:** Univariate analysis and multivariable logistic regression with clinical score and hospital complication

	Univariate analysis			Multivariate analysis		
	Odds Ratio	95% CI	*P* value	Odds Ratio	95% CI	*P* value
3‐month outcomes						
DCI	17.03	7.51‐38.61	<0.001	‐	‐	‐
Vasospasm	12.72	5.85‐27.69	<0.001	3.16	1.03‐9.70	0.045
SEBES (3‐4)	32.12	11.59‐160.36	<0.001	10.39	1.98‐54.64	0.006
HH (3‐5)	11.67	5.2‐26.21	<0.001	‐	‐	‐
WFNS (3‐5)	23.57	9.66‐57.52	<0.001	3.82	1.15‐12.67	0.029
GCS <15	7.53	3.64‐15.69	<0.001	‐	‐	‐
NPAN‐SAH	5.43	2.67‐10.71	<0.001	‐	‐	‐
12‐month outcomes						
DCI	26.28	9.68‐71.36	<0.001	‐	‐	‐
Vasospasm	100.22	13.16‐762.96	<0.001	31.69	3.65‐275.43	0.002
SEBES (3‐4)	53.45	16.01‐178.46	<0.001	10.01	1.87‐53.73	0.007
HH (3‐5)	14.17	5.55‐36.20	<0.001	‐	‐	‐
WFNS (3‐5)	51.65	17.44‐152.91	<0.001	5.15	1.34‐19.85	0.017
GCS <15	21.06	6.84‐64.86	<0.001	‐	‐	‐
NPAN‐SAH	12.21	4.03‐33.84	<0.001	‐	‐	‐

Abbreviations: GCS, Glasgow Coma Score; HH, Hunt‐Hess; SEBES, Subarachnoid hemorrhage Early Brain Edema Score; WFNS, World Federation of Neurosurgical Societies scale.

Although the clinical presentation and prognosis present significant difference between two bleeding patterns, the NPN‐SAH shows no such statistical significance in multivariate analysis, which may be as a result of the limited data size. In some cases, factors with important definition or clinical utility are also included, although the *P* value is more than 0.05, so that the nomogram may be a more reasonably available model.^21^, ^22^, ^23^ Thus, eventually, the high WFNS, high SEBES, and symptomatic vasospasm were determined as predictors. And the factor of NPAN‐SAH was included as a label, so that the nomogram can be reasonably applied in both types of AN‐SAH and be more clinically practical.

### 3.4 Multicollinearity test, nomograms, and validation of predictive accuracy for poor outcome

To establish a predictive model for clinical prognosis of AN‐SAH, we first tested the potential collinearity between variants within two models in the overall analysis. Variance inflation factor (VIF) < 3.5, tolerance > 0.3, minimum eigenvalue > 0.16, and condition index (CI) < 5 revealed no statistical significance in collinearity between variants in both of the present models.

**FIGURE 2:**
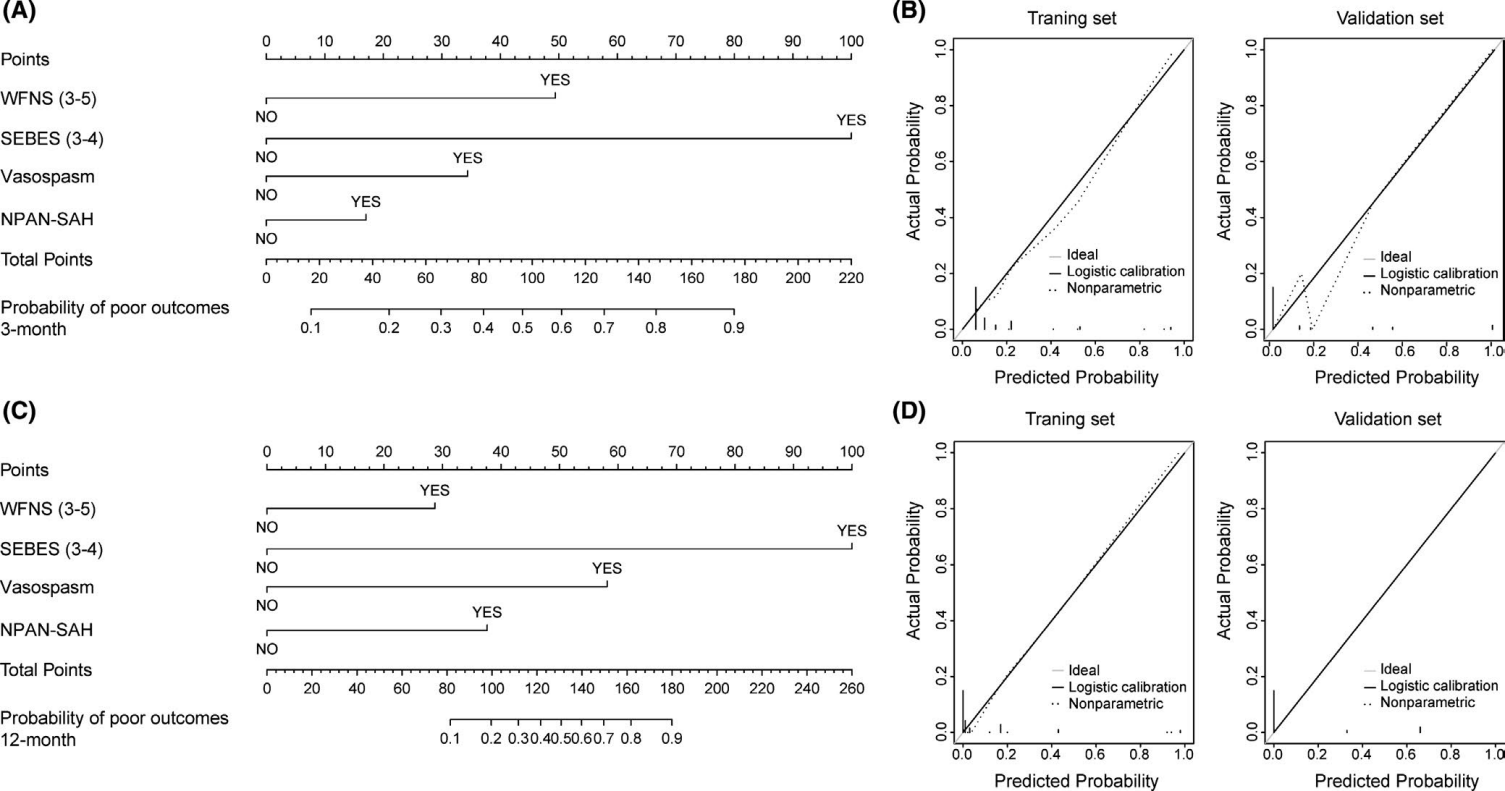
Nomogram for Post‐Discharge Outcome After 3‐Month and 12‐Month Follow Up. (A) To evaluate the probability of disability for an individual patient, review his/her clinical data and image features list in nomogram. Then, draw a vertical line from the feature status towards the points axis to obtain respective points based on each feature. Finally, draw a vertical line through the total points axis, according to the sum of the total score, which will intersect the probability of poor outcomes axis at the predicted probability. (B) Calibration curve for training cohort (left) and validation cohort (right). The grey line represents performance of ideal nomogram where the predicted probability perfectly corresponds to observed probability. Nomogram of 12‐month outcome (C) and its calibration curve (D)

Then, the patients were randomly divided into training cohorts and validation cohorts. There were no significant differences between the two groups of patients in demographic information and clinical characteristics (Table S2). Based on the multivariable regression analysis, we used nomograms to show the risk stratification for 3‐month and 12‐month disability with factors of WFNS, SEBES, and symptomatic vasospasm of the training cohort (Figure 2A and B). For an individual patient, risk factors contributed to respective points and the sum corresponded to the probability of 3‐month and 12‐month disability. The calibration curves suggested that the nomogram was relatively well calibrated and corresponded to prediction and observation. In the validation cohort, the calibration curve also showed minor discrepancies between observed and predicted probabilities (Figure 2C and D). The results showed that the AUC of the 3‐month nomogram of the training and validation cohorts are 0.752 and 0.975, respectively, while the AUC of the 12‐month nomogram are 0.931 and 0.986, respectively (Figure S2).

## 4 DISCUSSION

SAH is caused by ruptured intracranial aneurysms. Despite advanced therapy and critical care management, SAH is still companied with significant morbidity, mortality, and socioeconomic impact.^24^ This is not only caused by the hemorrhagic event, but further associated with the sequelae of the irritating blood by‐products, which can result in cerebral vasospasm and delayed cerebral ischemia (DCI). DCI has been reported to correlate with prognosis and neurological function after SAH.^25^ Several studies even applied micro‐CT, DSA to longitudinally monitor vasospasm and tried to reveal the progression after SAH.^26^ Besides, early brain injury (EBI) encompasses the entire brain injury occurring within 72 h after SAH, which is related to oxidative stress, neuroinflammation, neuronal death, blood‐brain barrier (BBB) damage, autophagy–apoptosis, and ferroptosis.^27^, ^28^, ^29^, ^30^ Although timely and effective treatment is implemented, the SAH‐induced neurological impairments are permanent in individual. Thus, it is important to monitor and prevent the occurrence of DCI.

To the best of our knowledge, this is the largest clinical retrospective study in AN‐SAH, and is the first to establish accurate predictive models paired with bleeding pattern. Consistent with the previous study, our study observed that patients with NPAN‐SAH presented significantly increased hospital complications and poorer functional outcomes compared with patients with PAN‐SAH. Previous study compared the occurrence of vasospasm between aSAH and PAN‐SAH. Notably, without any clinical significance in patients with PAN‐SAH, the incidence of vasospasm was 49.2%.^31^ The overall vasospasm rate of AN‐SAH in five studies was 16.3%. Meanwhile, the overall rate of DCI was 6.8%.^32^ In our present study, according to the bleeding pattern, the rate of vasospasm and delayed cerebral infarction in PAN‐SAH is 9.8% and 4.9%, respectively, while 55.1% and 30.3% in NPAN‐SAH.

An updated meta‐analysis, performed in 2019, suggested that differences in diagnostic strategy for identification of future vasospasm events and clinical status at admission may limit the skew of interpretation, which emphasized further analysis based on individual patient data to elucidate the clinical outcomes according to the hemorrhagic pattern in patients with AN‐SAH.^6^ In our present study, we demonstrated that symptomatic vasospasm is the more effective independent predictor than delayed cerebral infarction and other clinical complications. Consequently, patients with AN‐SAH require prompt augmentation of blood pressure or endovascular intervention if DCI occurs, and they should be monitored for vasospasm in the intensive care unit.^32^

In order to accelerate the turnover rate of patients, patients with AN‐SAH are often discharged for rehabilitation as soon as possible. Thus, the prognosis of patients at 3 months and 12 months varies widely, which emphasized the necessity of prognostic prediction in AN‐SAH patients. The present study focused on the prognosis of AN‐SAH patients from a larger sample size and presented a novel attempt to provide quantitative risk assessment of the poor outcome. We observed that the overall disability rate is 14.3% and 8.6% after recovery periods of 3 months and 12 months, respectively.

Current radiographic rating scales, the most widely used being the mFS grading system, are mainly focused on quantifying the amount of blood for the purpose of predicting vasospasm and DCI.^10^ In the term of clinical presentation, according to the International Cooperative Aneurysm Institute, the committee of experts assessed the findings that the level of consciousness is an important factor affecting the mortality and disability rate of patients. The most important factors affecting disability rate are hemiplegia and aphasia.^33^ Therefore, based on GCS, WFNS grading system was created and was selected in our present study. EBI is important as it reflects that the initial clinical presentation is the most important predictor of outcome.^34^ It is reported that SEBES is associated with clinical markers of EBI, such as HH and WFNS, and is an independent predictor of DCI and poor short‐term and long‐term clinical outcomes.^11^, ^35^ Furthermore, WFNS and SEBES simply reflect the aggravated brain injury related to the severity of the initial bleed, and exerted their predictive effects in AN‐SAH assessment than the other clinical scales. In our present study, the nomograms were constructed based on symptomatic vasospasm, WFNS, SEBES, and bleeding pattern to facilitate a quantitative assessment of disability for individual patients once their clinical and imaging data were available. Internal validation and performance statistics suggested that the current nomograms were predicting accurately. Since the factor of symptomatic vasospasm was included, the nomogram would allow for a longitudinal dynamic evaluation of prognosis, thereby making it possible to weigh the risk of various management options and recommend effective treatments at optimal time points.

There are several limitations in our study worth mentioning. First, the nomogram was established using retrospective data obtained from a single institution. Second, the outcome data of patients were recorded from outpatient and telephone interview at 3 and 12 months, for which there was referral bias as some patients were unacceptable due to unfavorable neurological status. Thus, a potential underestimation of poor prognosis might have influenced the overall estimate. Additionally, because of the relatively benign progression and limited cases, there is potential for minor bias to skew the interpretation. Although more data from other institutions are required for further validation of our nomograms, the individualized quantitative risk assessment using the present nomograms would be a more practical approach for predicting prognosis and counseling patients.

## 5 CONCLUSION

Symptomatic vasospasm, WFNS, SEBES, and bleeding pattern are independent predictors for 3‐month and 12‐month prognosis in patients with AN‐SAH. The nomograms could provide individualized prediction of post‐discharge outcomes of AN‐SAH patients, and represent a practical approach to disability assessment.

## Conflict of Interest

None.

## Author Contributions

JZ and SC: conception, supervision, and design of this article. AZ, ZZ, and WZ: data analysis and editing the manuscript. CL, YF, YL, and YL: data collection and patients’ follow‐up. XW and SM: establishment of predictive model. All authors contributed to the article and approved the submitted version.

## Ethical Approval

Clinical data analysis was approved by Institutional board of the Second Hospital affiliated to Zhejiang University (Clinical trial registration number: 2020269).

## Data Availability Statement

The supplementary material for this article can be found online. All processed data and R codes used in this study can be obtained from the corresponding author on reasonable request.

## Supporting information

Fig S1

Fig S2

Table S1

Table S2
