# Supplementary figures and images for: Development of a nomogram for predicting clinical outcome in patients with angiogram‐negative subarachnoid hemorrhage
Source: CNS Neurosci Ther. 2021 Jul 28;27(11):1339–47. doi: 10.1111/cns.13712 (PMC8504520; doi:10.1111/cns.13712)

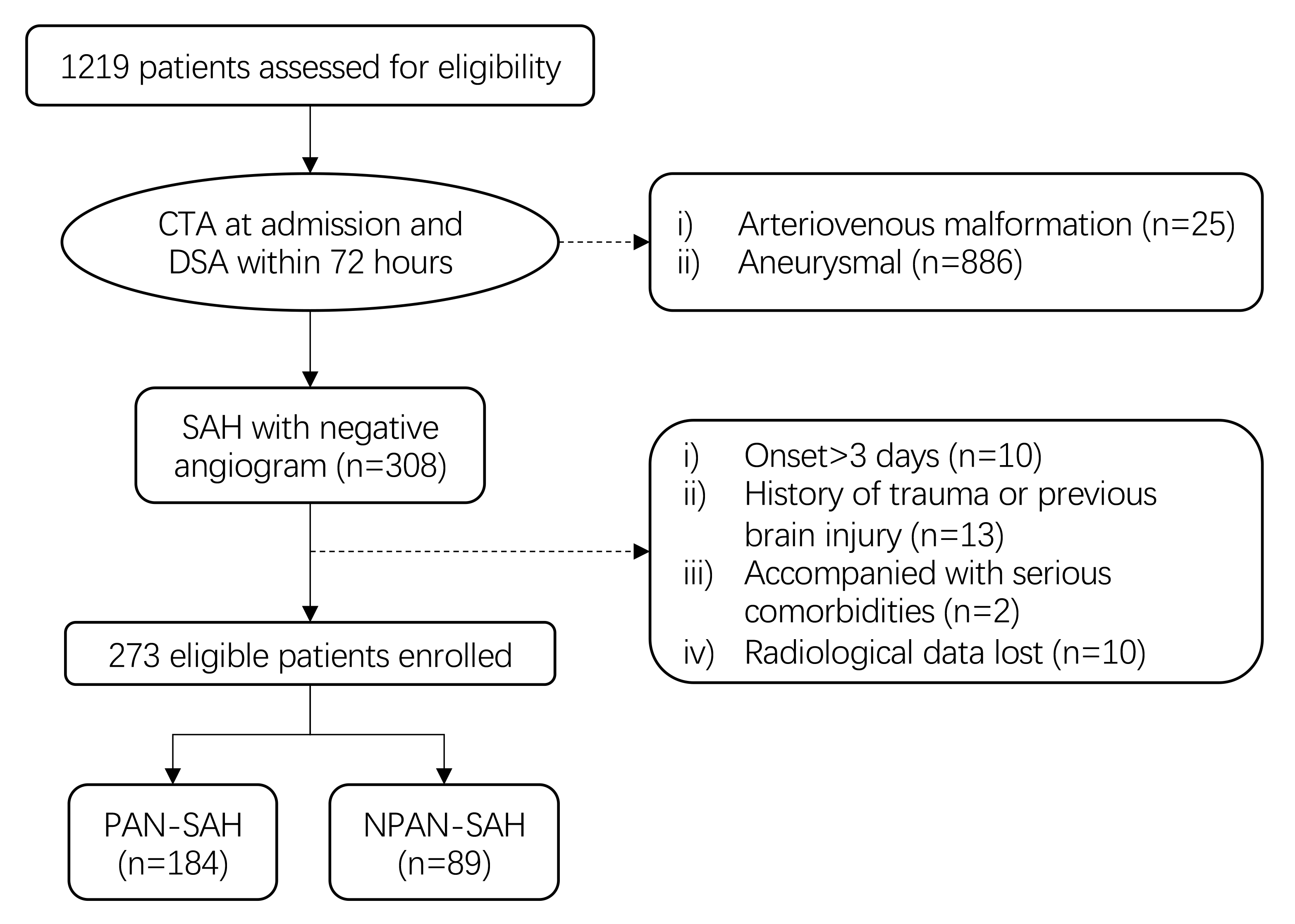

Supplement: Supplementary file 1 — Fig S1 [file CNS-27-1339-s002.tif]

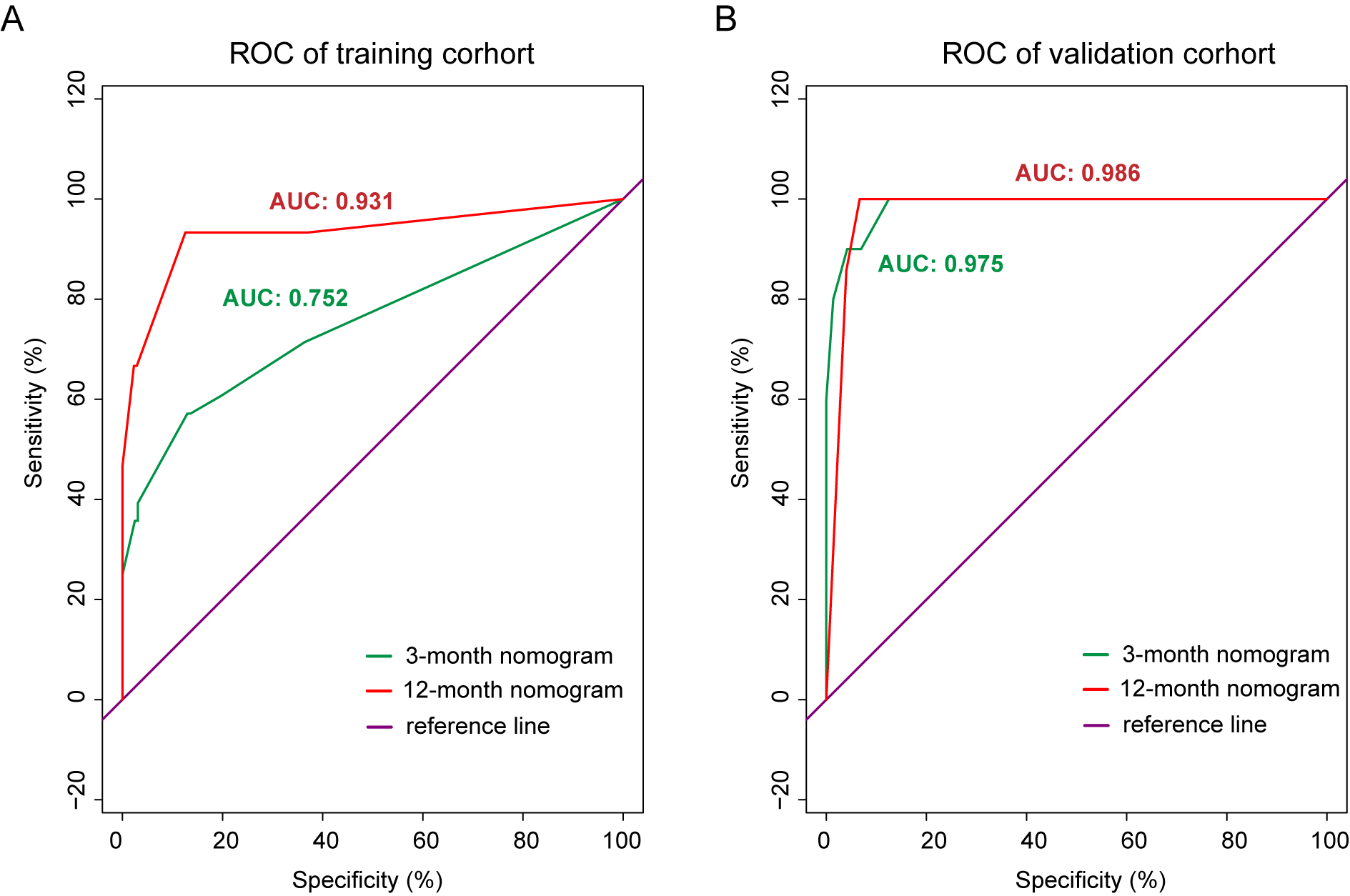

Supplement: Supplementary file 2 — Fig S2 [file CNS-27-1339-s003.tif]
