# Supplementary material for: Development of a nomogram for predicting clinical outcome in patients with angiogram‐negative subarachnoid hemorrhage
Source: CNS Neurosci Ther. 2021 Jul 28;27(11):1339–47. doi: 10.1111/cns.13712 (PMC8504520; doi:10.1111/cns.13712)
Supplement: Supplementary file 3 — Table S1 [file CNS-27-1339-s001.docx]

**Supplementary Table 1. Baseline characteristics of PAN-SAH and NPAN-SAH**

| Characteristics | PAN-SAH  No. of Patients (%) | NPAN-SAH  No. of Patients (%) | P value |
| --- | --- | --- | --- |
| Total number of patients | 184 (67.4%) | 89 (32.6%) |  |
| Gender (female) | 90 (48.9%) | 35 (39.3%) | 0.136 |
| Age (year, Mean±SD) | 55.7±10.7 | 57.4±11.8 | 0.368 |
| Drink | 69 (37.5%) | 34 (38.2%) | 0.911 |
| Smoke | 63 (34.2%) | 32 (36.0%) | 0.780 |
| Hypertension | 62 (34.2%) | 35 (38.2%) | 0.465 |
| Diabetes | 13 (7.1%) | 9 (10.1%) | 0.386 |
| BMI |  |  | 0.862 |
| I <18.5 | 8 (4.3%) | 3 (3.4%) |  |
| II 18.5-23.9 | 90 (48.9%) | 41 (46.1%) |  |
| III 24-27.9 | 79 (42.9%) | 40 (44.9%) |  |
| IV >28 | 7 (3.8%) | 5 (5.6%) |  |
| GCS |  |  | <0.0001 |
| I=15 | 155 (84.2%) | 53 (59.6%) |  |
| II 12-14 | 25 (13.6%) | 18 (20.2%) |  |
| III 9-11 | 2 (1.1%) | 3 (3.4%) |  |
| IV 3-8 | 2 (1.1%) | 15 (16.9%) |  |
| WFNS (3-5) | 6 (3.3%) | 24 (27.0%) | <0.0001 |
| HH (3-5) | 8 (4.3%) | 26 (29.2%) | <0.0001 |
| mFS (3-4) | 5 (2.7%) | 58 (65.2%) | <0.0001 |
| SEBES (3-4) | 2 (1.1%) | 15 (16.9%) | <0.0001 |
| IVH | 26 (14.1%) | 35 (39.3%) | <0.0001 |
| Symptomatic vasospasm | 18 (9.8%) | 49 (55.1%) | <0.0001 |
| Delayed cerebral infarction | 9 (4.9%) | 27 (30.3%) | <0.0001 |
| Rebleeding | 0 (0%) | 6 (6.7%) | <0.0001 |
| Encephaledema | 3 (1.6%) | 18 (20.2%) | <0.0001 |
| Seizure | 1 (0.5%) | 2 (2.2%) | 0.249 |
| Stay in hospital | 7.4±4.6 | 12.8±10.5 | <0.0001 |
| Poor 3-month outcome | 13 (7.1%) | 26 (29.2%) | <0.0001 |
| Poor 12-month outcome | 4 (2.2%) | 19 (21.3%) | <0.0001 |
| Mortality | 0 (0%) | 5 (5.6%) | 0.003 |
