# Supplementary material for: Development of a nomogram for predicting clinical outcome in patients with angiogram‐negative subarachnoid hemorrhage
Source: CNS Neurosci Ther. 2021 Jul 28;27(11):1339–47. doi: 10.1111/cns.13712 (PMC8504520; doi:10.1111/cns.13712)
Supplement: Supplementary file 4 — Table S2 [file CNS-27-1339-s004.docx]

**Supplementary Table 2. Clinical characteristics in training cohort and validation cohort.**

| Characteristics | Training cohort  No. of Patients (%) | Validation cohort  No. of Patients (%) | P value |
| --- | --- | --- | --- |
| Total number of patients | 190 | 83 |  |
| Gender (female) | 85 (44.7%) | 39 (47.0%) | 0.731 |
| Age (year, Mean±SD) | 55.7±10.7 | 57.4±11.8 | 0.368 |
| Drink | 70 (36.8%) | 33 (39.8%) | 0.647 |
| Smoke | 64 (33.7%) | 31 (37.3%) | 0.559 |
| Hypertension | 58 (30.5%) | 37 (44.6%) | 0.025 |
| diabetes | 16 (8.4%) | 5 (6.0%) | 0.494 |
| BMI |  |  | 0.003 |
| I <18.5 | 6 (3.2%) | 5 (6.0%) |  |
| II 18.5-23.9 | 86 (45.3%) | 44 (53.0%) |  |
| III 24-27.9 | 94 (49.5%) | 26 (31.3%) |  |
| IV >28 | 4 (2.1%) | 8 (9.6%) |  |
| GCS <15 | 28 (14.7%) | 36 (43.4%) | <0.0001 |
| WFNS (3-5) | 15 (7.9%) | 15 (18.1%) | 0.027 |
| HH (3-5) | 20 (10.5%) | 14 (16.9%) | 0.144 |
| mFS (3-4) | 41 (21.6%) | 23 (27.2%) | 0.271 |
| SEBES (3-4) | 8 (4.2%) | 8 (9.6%) | 0.079 |
| IVH | 40 (21.1%) | 21 (25.3%) | 0.438 |
| Symptomatic vasospasm | 47 (24.7%) | 20 (24.1%) | 0.910 |
| Delayed cerebral infarction | 22 (11.6%) | 14 (16.9%) | 0.235 |
| Rebleeding | 5 (2.6%) | 1 (1.2%) | 0.460 |
| Encephaledema | 15 (7.9%) | 5 (6.0%) | 0.585 |
| Seizure | 3 (1.6%) | 0 (0%) | 0.556 |
| NPAN-SAH | 64 (33.7%) | 25 (30.1%) | 0.563 |
| Poor 3-month outcome | 28 (14.7%) | 10 (12.0%) | 0.555 |
| Poor 12-month outcome | 15 (7.9%) | 7 (8.4%) | 0.880 |
| Mortality | 3 (1.6%) | 2 (2.4%) | 0.587 |
